# Initial evaluation of automated treatment planning software

**DOI:** 10.1120/jacmp.v17i3.6167

**Published:** 2016-05-08

**Authors:** Dawn Gintz, Kujtim Latifi, Jimmy Caudell, Benjamin Nelms, Geoffrey Zhang, Eduardo Moros, Vladimir Feygelman

**Affiliations:** ^1^ Department of Radiation Oncology Moffitt Cancer Center Tampa FL USA; ^2^ Canis Lupus LLC Merrimac WI USA

**Keywords:** automated treatment planning, treatment plan quality, head and neck treatment planning

## Abstract

Even with advanced inverse‐planning techniques, radiation treatment plan optimization remains a very time‐consuming task with great output variability, which prompted the development of more automated approaches. One commercially available technique mimics the actions of experienced human operators to progressively guide the traditional optimization process with automatically created regions of interest and associated dose‐volume objectives. We report on the initial evaluation of this algorithm on 10 challenging cases of locoreginally advanced head and neck cancer. All patients were treated with VMAT to 70 Gy to the gross disease and 56 Gy to the elective bilateral nodes. The results of post‐treatment autoplanning (AP) were compared to the original human‐driven plans (HDP). We used an objective scoring system based on defining a collection of specific dosimetric metrics and corresponding numeric score functions for each. Five AP techniques with different input dose goals were applied to all patients. The best of them averaged the composite score 8% lower than the HDP, across the patient population. The difference in median values was statistically significant at the 95% confidence level (Wilcoxon paired signed‐rank test p=0.027). This result reflects the premium the institution places on dose homogeneity, which was consistently higher with the HDPs. The OAR sparing was consistently better with the APs, the differences reaching statistical significance for the mean doses to the parotid glands (p<0.001) and the inferior pharyngeal constrictor (p=0.016), as well as for the maximum doses to the spinal cord (p=0.018) and brainstem (p=0.040). If one is prepared to accept less stringent dose homogeneity criteria from the RTOG 1016 protocol, nine APs would comply with the protocol, while providing lower OAR doses than the HDPs. Overall, AP is a promising clinical tool, but it could benefit from a better process for shifting the balance between the target dose coverage/homogeneity and OAR sparing.

PACS number(s): 87.55.D

## I. INTRODUCTION

Modern external beam radiotherapy features highly conformal, inversed‐planned treatment techniques such as IMRT and VMAT. However, even with these techniques, and factoring out variations in contouring,^1^, ^2^, ^3^, ^4^ the quality of treatment plans can vary greatly. Nelms et al.^(5)^ reported a study where different institutions were asked to produce a plan based on the same downloadable CT dataset with presegmented targets and normal structures. Also provided was a clear set of planning goals given as a list of metrics and per‐metric scoring methodology, producing a cumulative score called the Plan Quality Metric (PQM). This approach eliminated two major sources of uncertainty for a plan quality study: (i) variability in anatomy and contouring, and (ii) variability and subjectivity in the measure of plan quality. In this well‐controlled study, the results showed substantial variation in plan quality. Moreover, the variation was not readily attributable to any common technical factors such as delivery technique or treatment planning system (TPS) used. The authors concluded the variation was generally due to differences in “planning skills”. Echoing these findings, the current state of treatment planning was summarized by Moore et al.^(6)^ as “a very time‐consuming task with great output variability”.

One of the long‐established tenets in quality management—decreasing variability — is very much applicable to treatment planning, and is therefore one of the driving forces behind the development of more automated approaches. One proposed solution is based on the concept of machine learning. A database of previously accepted plans for a specific disease site is built. A new plan is supposed to achieve quality comparable to the previous cases with similar patient anatomy and objectives.^7^, ^8^, ^9^, ^10^ Another approach to partially automating the dose optimization process is implemented in the AutoPlanning (AP) software module, an option with Pinnacle v. 9.10 TPS (Philips Medical Systems, Fitchburg, WI). It requires no formal prior database of successful plans, but uses instead the iterative approach of progressive optimization.^(11)^ The concept is largely to capture the steps that a skilled human operator would take and then mimic them for a new patient.

In this paper, we perform an initial evaluation of this autoplanning approach by measuring the quality of the AP‐produced plans and comparing them directly to the quality of traditional human‐driven clinical plans created for the same datasets. To facilitate quantitative analysis of overall plan quality, we applied the PQM approach.^(5)^

## II. MATERIALS AND METHODS

### A. Autoplanning software

**Figure 1:**
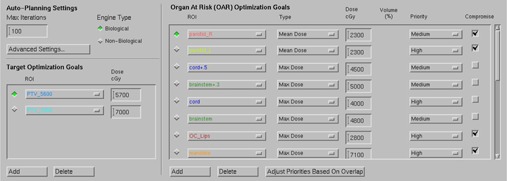
A partial Screenshot of the technique tab.

**Figure 2:**
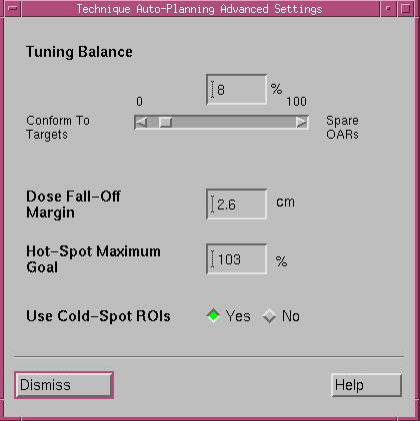
The Advanced Settings tab with the parameters used for all autoplans in this work.

The job of the previously described Pinnacle optimizer^(12)^ is to balance the competing objectives of target coverage and normal tissue sparing by minimizing the composite objective function. What is enhanced in AP is how the objectives are automatically created and used in iterative fashion. At the heart of the process is the concept called the “technique”. A technique includes a set of user‐supplied optimization goals, which follow the clinical dosimetry goals (Fig. 1). The target dose (left side of the figure) is defined by a single number (prescription dose). Additional user input is provided under Advanced Settings, where the maximum dose and a qualitative balance between target dose conformity and OAR sparing are set (Fig. 2). The Dose Fall‐Off Margin defines the width of an automatically created tuning ring structure around the PTV, across which the dose is supposed to decrease, ideally, from 100% to 50%. When Use Cold‐Spot ROIs box is checked, the AP engine identifies cold spots in the target and creates ROIs with corresponding objectives, to bring the dose up during the last three optimization loops.

The specific OAR goals are enumerated in the right panel. Their type could be Maximum or Mean dose, or a DVH point (volume at dose). As opposed to the weight factor from 0 to 100 used on the standard optimization tab, the user can qualitatively assign the relative importance of an individual goal (Priority) as High, Medium, or Low. It can be also specified as a hard constraint, but that option is seldom used as being too restrictive. The last column in Fig. 1 is Compromise. It is applicable to the situations when an OAR overlaps with a target. If the box is checked, it essentially means that the target owns the overlapping voxels and the OAR sparing could be compromised to achieve proper target coverage. That would be typically representative of a situation with a parallel OAR. For a serial OAR (e.g., the spinal cord), the box is left unchecked and the overlapping voxels are entirely owned by the OAR.

The software has an internal logic to check the level of overlap between a structure and a target and adjust the Priority accordingly. If a large portion of an OAR is inside the target volume and the Compromise box is checked, there is no point in having the priority set too high, and the software will automatically lower it according to the numerical level of overlap, based on 25% volume increments.

The core AP algorithm is based on the regional optimization concept introduced by Cotrutz and Xing,^(13)^ but is implemented based on the ROIs,^(11)^ as opposed to the original voxel‐based approach. It attempts to iteratively fine‐tune the target coverage and OAR sparing results by creating multiple additional structures, based both on the relative geometry of originally segmented regions of interest (ROI) and on the transient dose distributions transpiring during the optimization process. As those ROIs are created, they are automatically assigned dose‐volume objectives and added to the standard optimization tab, thus becoming an additional input to the optimizer. Those additional objectives are added to help meet high and medium priority goals.

This process of translating the clinical goals defined on the autoplanning page to the optimization objectives on the traditional IMRT tab^(12)^ is fairly complex. The starting objectives are not visible to the user, only the final set, after the autoplanning process is complete. The exact rules of the ROIs' and corresponding objectives' creation are proprietary. However, some observations can be made from a relevant plan example.

A planning target volume (PTV) prescription goal of uniform 70 Gy was translated into the minimum and maximum dose objectives for the whole target of 70.7 (101%) and 71.05 (101.5%) Gy, respectively. In addition, partial PTV volumes, apparently considered underdosed after the initial iterations, were assigned a 70 Gy minimum dose objective.

When the maximum dose goal for the OAR is specified, it translates into the corresponding maximum dose objective(s). What can be discerned from comparing the goal and objectives tabs, is that for a single goal, the software can create more than one objective, with different values and weights. For example, for the spinal cord planning volume at risk (PRV) clinical goal of 45 Gy maximum dose, two maximum dose objectives were created for the final optimization: 42.75 Gy (relative weight 100%) and 19.51 Gy (low relative weight of 0.125%). On the other hand, in order to implement the maximum dose goal of 28 Gy to the oral cavity, the algorithm simply applied the 28 Gy maximum dose objective to the portion of the OAR outside the PTV. However, the weight was kept low (0.2%), presumably since the objective was clearly unachievable due to the immediate proximity of the oral cavity to the primary PTV.

For one goal for the parotid mean dose, two different maximum equivalent uniform dose (EUD)^14^, ^15^ objectives were applied to the derived ROI — the part of the OAR outside the PTV. In general, if the “biological optimization” option is enabled, AP would use the EUD objectives whenever the mean dose goals are specified.

The AP technique can be saved and recalled later. The set of goals in the technique is typically (but not necessarily) accompanied by a previously established beam arrangement class solution, which is automatically applied when the technique is recalled. The process of AP commissioning consists largely of designing, by trial and error, of the technique(s) that produce desired outcome for a class of cases with similar clinical goals. While theoretically not requiring prior knowledge, the technique evaluation process is clearly influenced by the operator's perceptions of what a good plan should look like, and by the prior experience with similar plans.

### B. General evaluation methodology

#### B.1 Goals and scores in the plan quality algorithm

The plan quality scoring builds upon the previously established formalism^(5)^ which is based on defining a collection of specific metrics (which can be DVH points, conformality indices, etc.) and corresponding score functions for each. Each metric's score function translates the achieved value to a numerical score. The sum over all metric scores divided by the combined maximum possible constitutes a composite PQM (%), used as a proxy for the overall achieved plan quality. The individual score functions are generally designed to define a failure region (where the score is zero), a transition region between the minimally acceptable and the ideal achievements (where the score increases from zero to the maximum), and the region exceeding the ideal (where the maximum score is awarded). Once the quality algorithm is defined, the analysis is automated and devoid of observer bias. However, it is important to understand that the metric scores inevitably carry a degree of subjectivity when used in aggregate, for a composite PQM. It is fundamentally unavoidable when attempting to quantify the relative importance of different clinical priorities. On the other hand, an individual metric score (rendered as percentage of the maximum possible) is used to compare only that specific achievement for the single ROI across the plans, and thus is devoid of “relative importance” subjectivity.

The PQM algorithm used in this work is implemented in commercial PlanIQ software (v. 2.1, Sun Nuclear Corp, Melbourne, FL).

### C. Application to head and neck cancer treatment planning

#### C.1 Description of cases

To perform a challenging test of the AP algorithm, we applied it to some of the most dosimetrically difficult cases encountered in our practice — locoregionally advanced head and neck cancers. Ten consecutive, previously treated cases were selected according to the following criteria: all were treated with 6 MV VMAT beams for 35 fractions, with 70 Gy to the primary target (PTV_70) and simultaneously 56 Gy to the elective bilateral neck nodes (PTV_56); all patients were under the care of the same radiation oncologist and planned by the same dosimetrist. All original plans employed two or three full VMAT arcs and were designed for a Varian linear accelerator with a 120‐leaf Millennium multileaf collimator (Varian Medical Systems, Palo Alto, CA). The physician manually drew the primary gross tumor volume (GTV) and the elective nodes clinical tumor volume (CTV). The GTV was expanded uniformly by 5 mm to create the 70 Gy CTV. This was manually edited to remove bone, fascia, and air. Both CTVs were expanded uniformly by 3 mm to arrive at the corresponding planning target volumes (PTV). The primary (PTV_70) average target volume was 338±262 (1 SD) ${\text{cm}}^{3}$ with the range from 85 to $1035\;{\text{cm}}^{3}$. The bilateral elective nodes (PTV_56) had the average volume of $352\pm 94\;{\text{cm}}^{3}$, with the range from 182 to $490\;{\text{cm}}^{3}$.

#### C.2 AP technique development strategy

Although the ultimate intention was to evaluate VMAT planning, we originally attempted to use fixed‐gantry IMRT with nine beams to develop the set of AP dosimetric goals, as IMRT takes far less planning time. However preliminary trials indicated that it was not feasible to achieve plans of acceptable quality by the institutional standards, which was consistent with our previous manual planning experience. Therefore the AP techniques were developed with VMAT. To minimize the influence of delivery mechanical constraints on plan quality, all AP plans involved three full arcs with maximum delivery time of 140 s per beam and MLC motion constrained to 0.46 cm per 1° of gantry rotation.^(16)^ Allowing this ample delivery time during optimization provides the necessary freedom to the optimization algorithm, while the linac software usually finds a faster way to deliver the resulting plan.^(16)^ Full convolution calculation was performed after the 10th optimization iteration and the total number of iterations was limited to 100. The collimator was typically rotated $\pm 15^{\circ }$, except when a different angle was dictated by the target size. All plans were calculated on a $3\times 3\times 3\;{\text{mm}}^{3}$ grid with the Adaptive version of Pinnacle Collapsed Cone Convolution algorithm.^(17)^ Following the recommendations by Yartsev et al.^(18)^ for planning studies, the starting technique is presented in full detail in Table 1. This technique was used for the first series of autoplans (AP1) and was developed with vendor's assistance. The advanced tuning settings used for all APs are shown in Fig. 2.

Note that PTV_70 appears in this example twice (once as is, and once expanded by 1 mm to differentiate from the original). Initial experimentation has determined that one of the main AP challenges was to achieve adequate target coverage. Repeating the objective is just a practical way of instructing the optimizer to treat the target coverage with additional priority.

**Table 1:** The starting technique (AP 1).

*ROI*	*Goal Type*	*D (Gy)*	*V (%)*	*Priority*	*Compromise*
PTV_70	Target Dose	70		‐	‐
PTV_70+1 mm	Target Dose	70		‐	‐
PTV_56	Target Dose	57		‐	‐
Parotid (L and R)	Mean Dose	23		High	Yes
Parotid (L and R)	Max DVH	10	50	Medium	Yes
Cord	Max Dose	40		High	No
Cord+5 mm	Max Dose	50		High	No
Brainstem	Max Dose	48		Medium	No
Brainstem+3 mm	Max Dose	50		Medium	No
Oral cavity	Max Dose	28		High	Yes
Mandible	Max Dose	71		High	Yes
Inferior Pharyngeal Constrictor	Mean Dose	39		Medium	Yes
Superior/Middle Pharyngeal Constrictor	Mean Dose	51		Medium	Yes
Glottic and Supraglottic Larynx	Mean Dose	48		Low	Yes
Submandibular glands	Mean Dose	39		Low	Yes
Cerebellum	Max DVH	50	1	Low	Yes
Ring tuning structure around PTVs	Max Dose	71		Medium	Yes
Ring tuning structure around PTVs	Max DVH	56	25	Medium	Yes

The remaining techniques (APs 2‐5) were slight variations of the first one. The changes were primarily limited to attempts to improve target coverage and dose homogeneity. Since we require the entirety of the GTV to be covered by the prescription isodose, AP 2 and 3 included GTV as a separate target, with slightly higher dose goals. If GTV coverage were to improve, that would help to avoid excessive renormalization and thus improve dose homogeneity. In AP 4, a repeating goal was added for the secondary PTV (PTV_56 + 1 mm) in an attempt to achieve better coverage of the secondary target. For AP 5, in addition to having the GTV goals, two new tuning structures were created around the PTVs. The first one was a 1 cm expansion of PTV_70 and it was assigned a high priority maximum dose goal of 73.5 Gy. The second one was a part of PTV_56 at least 1 cm away from PTV_70. It was assigned a high priority maximum dose goal of 60 Gy. In the same technique, an additional larynx goal was introduced (maximum DVH dose of 35 Gy to 75% of the volume). All five techniques were applied to each of 10 cases, resulting in 50 autoplans.

#### C.3 Specific plan evaluation metrics

##### C.3.1 RTOG 1016 protocol acceptability

A basic goal of the evaluation is to determine if an automated planning routine can consistently and reliably produce “clinically acceptable” plans, which would define its success or failure in practice. It is important to note that “acceptable” (i.e., meeting minimal constraints) does not necessarily imply a plan of highest possible quality. The definition of acceptable is somewhat subjective and may vary from institution to institution and physician to physician. Therefore, we felt that it would be unfair to label the AP plans acceptable or unacceptable based on our internal criteria. Instead, for the initial screening, we adopted the consensus‐driven approach, from the RTOG protocol No. 1016.^(19)^ This particular H&N protocol uses the same primary and secondary dose levels (70 and 56 Gy) and has six dosimetric criteria that determine plan acceptability (Table 2). Of those six criteria, five deal with the target dose level and homogeneity, and one is concerned with the maximum dose to the spinal cord. The rest of the OAR sparing objectives are on the “best effort” basis, although recommendations on dose levels and priorities are provided.

There are slight differences with the protocol in how we apply the acceptability criteria. For the protocol, the first line in the table is automatically fulfilled if the plan is normalized as specified. We normalize out plans to cover 100% of the GTV with 100% of the prescription dose. Although this approach typically produces sufficient PTV coverage, compliance with the protocol had to be tested. The $0.03\;{\text{cm}}^{3}$ cold spot was first evaluated for the entire PTV_70. If failed, it was examined for the lesser volume as specified in the protocol, namely disregarding the PTV voxels residing closer than 8 mm from the skin.

**Table 2:** RTOG 1016 dosimetric acceptability criteria adapted to the current paper terminology.

	*Per Protocol*	*Variation Acceptable*	*Deviation Unacceptable*
Dose to 95% of PTV_70	70 Gy	None	None
Minimum dose to 0.03 cc inside PTV_70 and ≥8 mm inside the skin	66.5 Gy	>66.5 but >63 Gy	≤63 Gy
Maximum dose (>1 cc “hot spot”) in PTV_70	<77Gy	>77 but <82 Gy	>82 Gy
Maximum dose (>1 cc “hot spot”) outside the PTVs	>82 Gy	74‐77 Gy	>77 Gy
Dose to 95% of PTV_56	56 Gy	≥45 but <56 Gy	<45 Gy
Max dose (0.03 cc hot spot) to Cord+5 mm	≤50 Gy	>50 but ≤52 Gy	>52 Gy

##### C.3.2 Institutional plan quality scores

**Figure 3:**
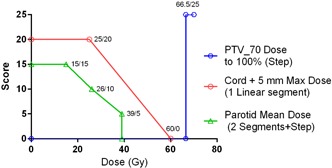
Examples of the score functions, illustrating how different numbers of value/score pairs in Table 3 (reproduced on the graphs) define functions of different shapes.

The individual metric score functions used to calculate the plan quality scores are presented in Table 3, on the left. The minimum number of points necessary to describe every function is given. A step function is thus defined by one value/score combination, a single‐slope linear function by two, and two linear segments with different slopes by three. Examples of how the value/score pairs from Table 3 define the shape of the score function for each of the three scenarios above are given in Fig. 3.

The score functions reflected the target and OAR goals routinely employed in our clinic and recorded on the formalized objective sheets. They were defined prior to commencement of the AP evaluation. The OAR score values are based on biological endpoints^20^, ^21^, ^22^, ^23^ and attempt to capture the physician's perception of the relative importance of different dose goals. Taking the parotid gland as an example, the maximum available score is 15, relative to the target coverage maximum score of 25. This reflects the facts that curing cancer is considered more important than preserving salivary function and that the parotids are not the only saliva‐producing glands. The parotid dose/score points are based on the simplified version of the normal tissue complicated probability (NTCP) curve from Dijkema et al.^(23)^ who plotted the probability of saliva flow ratio at 1 year reduced to <25% against the mean parotid dose. As seen in Table 3, 15 points is awarded for the mean dose of 15 Gy (∼5% NTCP), 10 points for 26 Gy (∼25% NTCP), and 5 points for 39 Gy (≤50 NTCP). No points are awarded above 39 Gy. Thus the AP5 average mean parotid dose PQM score in Table 3 (74.8%) is equivalent to 0.748×15=11.2 points. From the plot in Fig. 3, it translates back into the absolute dose of 23.7 Gy. Similar calculations can be easily performed, if desired, for every objective in Table 3.

Table 3 is divided into four parts. The first three list the objectives that were used for planning and evaluation, grouped into Target Coverage, Target Dose Homogeneity, and OAR Sparing categories. The last group of indices, Excluded From Scoring, contains two entries that were not a part of the original plan evaluation and comparison, but were deemed worthwhile to investigate after the fact. Total irradiated volume at 73.5 Gy (105% of prescription) is self‐explanatory. The Conformation Number (CN)^(24)^ is one of several ways^(25)^ to quantify the reference isodose volume (70 Gy) conformality to the target (PTV_70). It is defined as

(1) $$CN=\frac{V_{T,ref}}{V_{T}}\times \frac{V_{T,ref}}{V_{ref}}$$

where $V_{T,ref}$ is the volume of target receiving a dose equal to or greater than the reference dose, $V_{T}$ is the target volume, and $V_{ref}$ is the volume receiving a dose equal to or greater than the reference dose.

For each patient, the overall PQM score was recorded for the original plan and the test plans generated from five different AP templates for this study. This means that overall 60 plans were generated and analyzed. An AP template with the highest average composite score across 10 patients was selected as the best one, and the plans it produced were compared to the original human‐driven plans (HDPs) in greater detail, at the individual goals level.

Since, following the PQM method, all results were recorded as a percentage of the predefined maximum possible score (whether combined or for individual objectives), a higher number always means a more desirable result, whether the context is covering or sparing. The only exception is the last two lines in Table 3, which contain absolute values. Not every OAR was segmented and evaluated for every plan (see the last column in Table 3 showing in parenthesis the number of cases where each objective was scored). However since for each patient the same set of OARs was evaluated, the cumulative score comparison between the different plans for the same case is still valid.

**Table 3:** The left half of the table lists all the planning and evaluation objectives: the ROI, the type of objective (dose to volume, volume at dose or other), and the goal values with associated scores (e.g., the score functions). The right half presents the resulting descriptive statistics comparing the human‐driven plan (HDP) and the overall highest‐scoring PQM autoplan (AP 5). The last column lists the p‐value for the Wilcoxon test (number of pairs).

					*Mean PQM* ±1 SD *(%)*		*Range*		
*RO1*	*Objective Type*	*Point 1/Score*	*Point 2/Score*	*Point 3/Score*	*HDP*	*AP 5*	*HDP*	*AP 5*	*P (N)*
*Target Coverage*									
PTV_70	V@70 Gy (%)	95/25			100	100	‐	‐	NA(10)
PTV_70	D (Gy) to 100%	66.5/25			70±48.3	70±48.3	0‐100	0‐100	NA(10)
PTV 56	V@56 Gy (%)	95/25			100	100	‐	‐	NA(10)
PTV56	D (Gy) to 100%	53.2/25			70±48.3	20±42.2	0‐100	0‐100	NS(10)
*Dose Homogeneity*									
‐	Max Dose (Gy)	73.5/20	74.9/0		82.1±32.5	23.2±34.1	0‐100	0‐100	0.008(10)
PTV70	V@73.5 Gy (%)	1/25	15/0		100	71.3±37.7	‐	0‐100	0.03(10)
*Normal Tissue Sparing*									
‐	Max Dose Location	GTV/10	PTV_70/5	PTV_56/1	65±24.2	40+21.1	50‐100	0‐50	NA
Cord+5 mm	Max D (Gy)	25/20	60/0		21.5±5.4	32.8±15.1	15.1‐34.2	20.1‐60.8	0.018(10)
Cord+5 mm	Mean D (Gy)	26/5			90±31.6	90±31.6	0‐100	0‐100	NA(10)
Cord+5 mm	V@30 Gy (%)	45/5			90±31.6	90±31.6	0‐100	0‐100	NA(10)
Cord+5 mm	V@40 Gy (%)	10/5			100	100	‐	‐	NA(10)
Brainstem+3 mm	Max D	25/20	54/0		70.5±20.8	89.7±20.8	41.2‐100	37.3‐100	0.04(10)
Brainstem+3 mm	V%55 Gy (cc)	2.7/5			100	100	‐	‐	NA(10)
Brainstem+3mm	V@60 Gy (cc)	0.9/5			100	100	‐	‐	NA(10)
Brainstem+3 mm	Mean D	36/5			100	100	‐	‐	NA(10)
Brain	Max D (Gy)	20/5	50/0		22	22.3	‐	‐	NA(2)
Brain	V@50 Gy (%)	5/5	10/0		100	100	‐	‐	NA(2)
Parotids	Mean D (Gy)	15/15	26/10	39/5	67.0±22.9	74.8±21.1	0‐100	0‐100	<0.001(20)
SMGs	Mean D (Gy)	15/15	39/10	45/5	19.2±27.7	19.6±28.9	0‐67	0‐72.1	NS(11)
Mandible	Max D (Gy)	50/10			0	0	‐	‐	NA(10)
Mandible	V@70 Gy (%)	7/5			80.0±42.2	80.0±42.2	0‐100	0‐100	NA(10)
Mandible	V@60 Gy (%)	35/5			80.0±42.2	80.0±42.2	0‐100	0‐100	NA(10)
Mandible	V@50 Gy (%)	62/5			80±42.2	80±42.2	0‐100	0‐100	NA(10)
GSL	Mean D (Gy)	20/15	40/10	51/0	26.1±25.4	34.0±31.7	0‐69.7	0‐72.2	NS(8)
GSL	V@35 Gy (%)	79/5			50.0±53.5	75.0±46.3	0‐100	0‐100	NS(8)
GSL	V@45 Gy (%)	45/5			62.5±51.8	50±53.5	0‐100	0‐100	NS(8)
GSL	V@55 Gy (%)	32/5			50.0±53.5	37.5±51.8	0‐100	0‐100	NS(8)
GSL	V@65 Gy (%)	22/5			37.5±51.8	37.5±51.8	0‐100	0‐100	NS(8)
OC_Lips	Mean D (Gy)	20/15	32/10		40.1±48.8	49.8±42.2	0‐100	0‐100	NS(10)
SPC	Mean D (Gy)	20/15	51/5	54/0	14.5±18.6	16.4±19.5	0‐43.9	0‐39.5	NS(8)
MPC	Mean D (Gy)	20/15	51/5	54/0	15.6±19.6	19.7±24.7	0‐41.4	0‐50.3	NS(8)
IPC	Mean D (Gy)	20/15	51/5	54/0	40.7±24.2	48.5±26.6	0‐67.4	0‐72.5	0.016(10)
IPC	V@40 Gy (%)	65/5			55.6±52.7	88.9±33.3	0‐100	0‐100	NS(10)
IPC	V@50 Gy (%)	47/5			70.0±48.3	80.0±42.2	0‐100	0‐100	NS(10)
IPC	V@60 Gy (%)	11/5			40.0±51.6	50.0±52.7	0‐100	0‐100	NS(10)
Cerebellum	V@50 Gy (cc)	0/20	1/10	5/0	99.8±0.5	99.2±1.5	98.7‐100	96.8‐100	NS(10)
*Excluded From Composite Scoring*									
PTV70	CN 70 Gy	‐			0.807±0.06	0.818±0.04	0.669‐0.912	0.747‐.882	NS(10)
	Irradiated V@73.5 Gy (cc)	‐			0.6±1.2	63.1±210	0‐3.9	1‐501.8	0.004((10)

SMG = submandibular gland; GSL = glottic and supraglottic larynx; OC_Lips = oral cavity and lips; S/M/IPC = superior/middle/inferior pharyngeal constrictor; CN = Conformation Number (see Methods for definition); NS = non‐significant.

##### C.3.3 Statistical analysis

The goal was to ascertain if there was a statistically significant difference between the scores for the HDPs and APs aggregated across the patient population. Both total and individual objectives' scores were compared. The nonparametric Wilcoxon matched‐pairs, signed‐rank test was used with p‐value below 0.05 considered significant. This test is used to determine if the medians of two paired distributions are statistically different when the distributions cannot be assumed Gaussian. The analysis was implemented in GraphPad Prism v. 6.0 software (GraphPad Software, La Jolla, CA).

## III. RESULTS

### A. RTOG 1016 acceptability

For both HDPs and APs, 95% percent of both PTV_70 and PTV_56 volumes were covered by the respective prescription doses with no deviations. Likewise, the cord constraint was always fulfilled. With the rest of the acceptability criteria, for nine out of ten patients, all plans either fully complied with the protocol or exhibited only minor deviations. With one patient, no plan was able to keep the 0.03 cc cold spot above 63 Gy for the original PTV_70 and only the HDP achieved a minor deviation with PTV_70 cropped 8 mm away from the skin (64.8 Gy). The AP PTV_70 cold spots ranged from 59.7 to 62.1 Gy. For all other patients, the cold spot was RTOG‐acceptable, even with the full PTV_70.

### B. Institutional plan quality scores

The descriptive statistics of the composite PQM are presented in Table 4 for the HDPs and five APs. The technique modification efforts described in Methods had some desired effects. In AP4, adding the second, nearly identical PTV_56 objective increased the number of cases where the dose to 100% of PTV_56 was at or above 53.2 Gy from 3 to 7 out of 10. It is the same number as for the HDP (although not in all the same cases). The plans generated by technique No. 5 (AP 5) had the highest average PQM among the APs and that technique was selected for detailed evaluation. This improvement in the AP composite score is attributable to the success of additional operator‐added tuning structures in improving dose homogeneity. Combining the scores for the goals of maximum dose and PTV_70 volume at or above 73.5 Gy, the average for AP 5 stands at 47.2%, with the next best one, AP 2, at 35.4%. For comparison, the HDP achieved 91.1% on average.

**Table 4:** Descriptive statistics (N=10 patients) for composite plan quality metrics (PQM) for the original human–driven plan (HDP) and five autoplans (APs 1‐5).

	*Composite PQM (%) Plan*					
	*HDP*	*AP 1*	*AP 2*	*AP 3*	*AP 4*	*AP 5*
Mean	65.2	56.0	58.7	58.2	59.6	59.9
SD	10.9	8.7	7.7	7.8	10.9	9.1
Min	45.6	44.9	44.8	47.6	46.8	44.8
Max	77.7	72.0	72.5	68.8	73.6	75.1

The median composite PQM scores are statistically different between the HDPs (65.4%±10.9% (1 SD), and AP 5 plans (59.9%±9.1%). Wilcoxon test p‐value for median difference was 0.027. Note that the relatively low reported scores are indicative not of the poor quality plans but rather of the high evaluation bar. For example, to score 100%, the mean parotid dose must be no more than 15 Gy, as opposed to the RTOG 1016 26 Gy guideline.^(19)^ The detailed comparison at the individual objectives level is presented in Table 3, on the right‐hand side.

In terms of target coverage, as described above, every plan delivered at least 70 Gy to 95% of the PTV_70 volume. Similarly, 95% of PTV_56 volume was always covered by the 56 Gy isodose. The minimum PTV dose was below desired 66.5 Gy (95% of prescription) in the same three cases for both the APs and the HDPs. This does not contradict the previously described analysis by the RTOG 1016 criteria, since the minimum dose here is defined for a single voxel. Finally, there was a difference, albeit not statistically significant, in the minimum dose (single voxel) to PTV_56. Three HDPs and seven APs failed to reach the 53.2 Gy (95% of prescription) goal. However, for one patient the HDP failed and the AP did not.

Although adding a tuning structure (in AP 5) has improved dose homogeneity some, the AP results in this subgroup are still inferior to the HDPs. The maximum dose (single voxel) was lower with high statistical significance (p=0.008) for the HDPs. For those, only one case had a hot spot above 107% of prescription (0 score), and six cases recorded ≤105 of prescription (100% score). At the same time, six of the APs failed to maintain the maximum dose below 107% of the prescription, and only one had the hot spot under 105%. Also statistically significant (p=0.03) was the difference for the PTV volume at or above 73.5 Gy (105% of prescription). In all HDPs, the volume did not exceed $1\;{\text{cm}}^{3}$ (100% score). Four of the APs achieved the same result, while one had the ≥105% dose volume in the PTV above $15\;{\text{cm}}^{3}$ (zero score), with the remaining five falling in between 1 and $15\;{\text{cm}}^{3}$. While not a part of the tabulated composite score, the total volume irradiated to at least 73.5 Gy exhibited highly statistically significant difference between the median values of the HDPs and APs (p=0.004). In the HDPs, it was kept consistently low ($0-3.9\;{\text{cm}}^{3}$), while for the APs it varied widely from 1 to $501.8\;{\text{cm}}^{3}$. Once again, this does not contradict the RTOG 1016 acceptability analysis since the hot spot here is defined more stringently: at a lower level (75 vs. 82 Gy) and for a single voxel, as compared to $1\;{\text{cm}}^{3}$ in the protocol. For the HDPs, the hot spot was always located either in the GTV (3 cases) or PTV_70. For the APs, the hot spot was located in PTV_70 eight times, and twice elsewhere. Interestingly, despite the difference in dose homogeneity, the conformity number between PTV_70 and the 70 Gy dose volume was essentially the same between the HDPs and APs.

While the dose homogeneity scores favor the HDPs over the APs, the trend is largely reversed for OAR sparing. Both the cord and the brain stem maximum dose (single voxel) PQM scores are statistically significantly higher (meaning lower dose) for the APs (Table 3). The parotid mean dose score is also higher for the APs with high statistical significance (p<0.001). The last OAR with a statistically significant advantage for the APs was the inferior pharyngeal constrictor.

**Figure 4:**
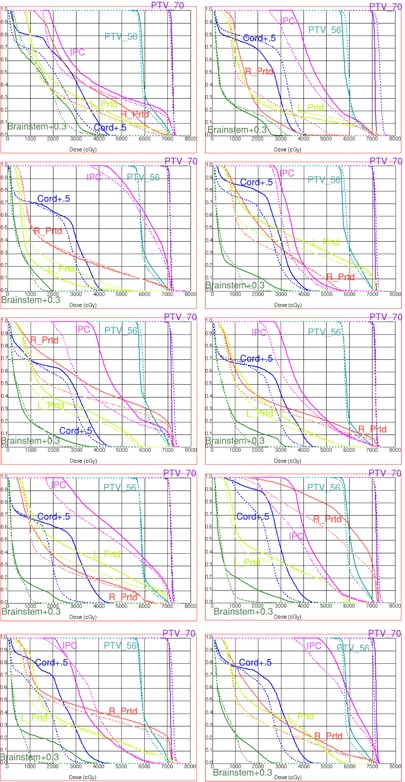
Representative dose‐volume histograms for all cases. Solid lines represent HDP and dashed AP 5. Structure names are color‐coded on the graphs (PTV 70 and 56 Gy, Left and Right Parotids, Cord+5 mm, Brainstem+5 mm, and Inferior Pharyngeal Constrictor (IPC)).

To facilitate a more familiar mode of the data review, the parameters found to have statistically significant differences between the HDPs and APs are recast in Table 5 in terms of original dose‐volume points, as opposed to the normalized scores in Table 3. In addition, the DVH curves for those structures, along with the targets, are presented in Fig. 4 for all 10 cases. It is immediately clear that, when the goal is to minimize the mean (EUD) OAR dose (the parotids and IPC), the corresponding AP DVH curves run consistently below the HDP ones. On the other hand, when only the maximum dose goal is specified (the cord and brainstem), the relative shape of the DVH curves varies.

The difference between the HDP and AP scores did not achieve statistical significance for the remaining 26 normal tissue sparing objectives. Of those, 24 scores were equally divided in being either equal or higher for the APs, and two were in favor of the HDPs (percentages of larynx volume at or above 45 and 55 Gy). However the scores for the mean larynx dose and the volume at or above 35 Gy were higher for the APs. Incidentally, those were the two parameters that showed improvement after modifying the larynx goal in AP5.

**Table 5:** Select parameters from Table 3 presented as original dose‐volume data. High–dose homogeneity and OAR sparing parameters that are statistically significantly different between the HDP and AP5 are summarized here.

		mean±1 SD		*Range*	
*ROI*	*Objective Type*	*HDP*	*AP 5*	*HDP*	*AP 5*
‐	Max Dose (Gy)	73.7±0.5	75.5±1.2	73.0‐74.2	73.4‐77.7
PTV_70	V@73.5 Gy (%)	0.2±0.3	8.1±14.5	0.0‐1.0	0.0‐47.6
Cord+5 mm	Max D @@(Gy)	44.8±1.3	42.0±3.3	41.6‐46.4	35.0‐45.2
Brainstem+3 mm	Max D	33.1±7.1	24.4±8.7	19.5‐42.2	17.2‐43.4
Parotids	Mean D@@(Gy)	26.7±10.3	23.7±7.8	10.4‐60.6	14.7‐51.7
IPC	Mean D@@(Gy)	46.7±9.4	42.5±9.1	35.3‐61.8	32.9‐59.8

## IV. DISCUSSION

### A. Differences between the auto‐ and human‐driven plans

To deliver a desired dose to the target, a certain amount of radiative energy has to cross its surface, depending on the size. That energy fluence cannot be reduced. What can be influenced is the pattern in which this energy is delivered. The process of treatment planning attempts to find a pattern that strikes a reasonable compromise between the three competing priorities: target coverage, target dose homogeneity, and sparing of the adjacent OARs. A TPS optimizer is essentially a black box to the user. A set of numbers (objectives) is provided as an input, resulting in an output (a dose distribution typically further reduced to a set of DVHs). Ideally, the inputs should be intuitive, and the black box should be sensitive and linear (e.g., allow for an easy shift in the priorities and direct proportionality between the change in the input and corresponding output).

The input parameters to Pinnacle AP are rather intuitive. They are for the most part clinical dose‐volume goals, with a few “tricks of the trade” applied occasionally (see AP technique development strategy section in Methods). However, what we found in this work is that the AP algorithm seems to have a natural tendency to favor OAR sparing over target coverage and closely related target dose homogeneity. The higher composite PQM scores for the human‐driven plans are a reflection of the institutional priorities for the H&N plans built into the scoring algorithm, as we put a premium on target dose homogeneity. Other institutions or physicians may be willing to accept larger hot spots in exchange for better OAR sparing. If one is satisfied with meeting the basic RTOG 1016 hot and cold spot criteria, the AP OAR sparing results are actually preferable. What AP is apparently lacking, at least at our level of experience, is an easy tool to move the balance between dose homogeneity and OAR sparing. While problematic with our approach to the conventional H&N planning, the relative ease of achieving a lower dose to the OARs at the expense of higher hot spots could be beneficial, for example, for radiosurgery‐type treatments, where the target dose inhomogeneity is mandated rather than discouraged.^(26)^

The evaluation was done for the highest H&N primary dose level employed in our clinic (70 Gy). Since the OAR absolute dose goals stay the same with the lower primary dose (e.g. 66 or 60 Gy), satisfactory performance is expected in those cases.

### B. Do the autoplans drive every goal to its achievable limit?

Perhaps the most intriguing feature of the Pinnacle autoplanning engine is that it is designed, according to the vendor, to push the dosimetric indices beyond the specified goals and towards achievable limits. To definitively verify this claim, one needs to formally demonstrate that the resulting plans are Pareto‐optimal (i.e., no objective can be improved upon without simultaneously sacrificing at least one other).^(27)^ This task is formidable^(28)^ even with the proposed reduction in the number of evaluated plans,^29^, ^30^ and typically five or fewer dose objectives were considered in the cited references. Therefore Pareto analysis, particularly exhaustive in terms of the structures, was beyond the scope of the current practical study. However, using a composite plan quality metric encompassing challenging evaluation scores is a step in the right direction. Applying it to select the best technique helps to find a balanced solution, where a dosimetric improvement for one structure is more likely to result in deterioration for the other(s). Furthermore, achieved dose values for certain OARs were routinely below the specified goals. This is consistent with the claimed algorithm behavior, although does not prove it. Further studies, with different methodology, are needed to find out if the dose homogeneity could be improved without sacrificing OAR sparing.

A novel automated treatment planning algorithm was evaluated on 10 realistic, challenging VMAT head and neck cases in comparison with the traditional human‐driven plans used for actual treatments. Side‐by‐side comparisons were performed using objective, formalized plan quality metrics. Human‐driven plans provided more homogeneous dose distributions and the autoplans excelled at limiting the OAR doses, while still conforming to the relevant RTOG dose homogeneity requirements. The DVHs for some OARs were driven by AutoPlanning beyond the specified goals, but overall this study was not equipped to probe if the software produced Pareto‐optimal plans. AutoPlanning appears to be a robust clinical tool, but it would be, in our opinion, beneficial to improve the user controls for rebalancing the optimization compromise away from OAR sparing and towards dose homogeneity, to better fit the range of preferences of different clinicians.

## ACKNOWLEDGMENTS

We are grateful to Philips for providing the AutoPlanning system for evaluation, and to K. Bzdusek and F. Nunez for invaluable help with the original technique setup. This work was supported in part by a grant from Sun Nuclear Corp.

## COPYRIGHT

This work is licensed under a Creative Commons Attribution 4.0 International License.

## Supporting information

Supplementary Material

Supplementary Material

Supplementary Material

Supplementary Material

## References

[1] Li XA , Tai A , Arthur DW , et al. Variability of target and normal structure delineation for breast cancer radiotherapy: an RTOG multi‐institutional and multiobserver study. Int J Radiat Oncol Biol Phys. 2009;73(3):944–51. 19215827 10.1016/j.ijrobp.2008.10.034 PMC2911777

[2] Hong TS , Tomé WA , Harari PM . Heterogeneity in head and neck IMRT target design and clinical practice. Radiother Oncol. 2012;103(1):92–98. 22405806 10.1016/j.radonc.2012.02.010 PMC3694728

[3] Yamamoto M , Nagata Y , Okajima K , et al. Differences in target outline delineation from CT scans of brain tumours using different methods and different observers. Radiother Oncol. 1999;50(2):151–56. 10368038 10.1016/s0167-8140(99)00015-8

[4] Nelms BE , Tome WA , Robinson G , Wheeler J . Variations in the contouring of organs at risk: test case from a patient with oropharyngeal cancer. Int J Radiat Oncol Biol Phys. 2012;82(1):368–78. 21123004 10.1016/j.ijrobp.2010.10.019

[5] Nelms BE , Robinson G , Markham J , et al. Variation in external beam treatment plan quality: an inter‐institutional study of planners and planning systems. Pract Radiat Oncol. 2012;2(4):296–305. 24674168 10.1016/j.prro.2011.11.012

[6] Moore KL , Kagadis GC , McNutt TR , Moiseenko V , Mutic S . Vision 20/20: Automation and advanced computing in clinical radiation oncology. Med Phys. 2014;41(1):010901. 24387492 10.1118/1.4842515

[7] Petit SF , Wu B , Kazhdan M , et al. Increased organ sparing using shape‐based treatment plan optimization for intensity modulated radiation therapy of pancreatic adenocarcinoma. Radiother Oncol. 2012;102(1):38–44. 21680036 10.1016/j.radonc.2011.05.025 PMC3578316

[8] Wu B , Ricchetti F , Sanguineti G , et al. Data‐driven approach to generating achievable dose‐volume histogram objectives in intensity‐modulated radiotherapy planning. Int J Radiat Oncol Biol Phys. 2011;79(4):1241–47. 20800382 10.1016/j.ijrobp.2010.05.026

[9] Wu B , Ricchetti F , Sanguineti G , et al. Patient geometry‐driven information retrieval for IMRT treatment plan quality control. Med Phys. 2009;36(12):5497–505. 20095262 10.1118/1.3253464

[10] Moore KL , Brame RS , Low DA , Mutic S . Experience‐based quality control of clinical intensity‐modulated radiotherapy planning. Int J Radiat Oncol Biol Phys. 2011;81(2):545–51. 21277097 10.1016/j.ijrobp.2010.11.030

[11] Xhaferllari I , Wong E , Bzdusek K , Lock M , Chen J . Automated IMRT planning with regional optimization using planning scripts. J Appl Clin Med Phys. 2013;14(1):176–91. 10.1120/jacmp.v14i1.4052 PMC5714048 23318393

[12] Bedford JL , Childs PJ , Warrington AP . Verification of inverse planning and treatment delivery for segmental IMRT. J Appl Clin Med Phys. 2004;5(2):1–17. 10.1120/jacmp.v5i2.1975 PMC5723460 15738908

[13] Cotrutz C and Xing L . IMRT dose shaping with regionally variable penalty scheme. Med Phys. 2003;30(4):544–51. 12722806 10.1118/1.1556610

[14] Thieke C , Bortfeld T , Niemierko A , Nills S . From physical dose constraints to equivalent uniform dose constraints in inverse radiotherapy planning. Med Phys. 2003;30(9):2332–39. 14528955 10.1118/1.1598852

[15] Lee TF , Ting HM , Chao PJ , et al. Dosimetric advantages of generalised equivalent uniform dose‐based optimisation on dose‐volume objectives in intensity‐modulated radiotherapy planning for bilateral breast cancer. Br J Radiol. 2012;85(1019):1499–506. 23091290 10.1259/bjr/24112047 PMC3500793

[16] Feygelman V , Zhang GG , Stevens CW . Initial dosimetric evaluation of SmartArc — a novel VMAT treatment planning module implemented in a multi‐vendor delivery chain. J Appl Clin Med Phys. 2010;11(1):99–116. 10.1120/jacmp.v11i1.3169 PMC5719766 20160702

[17] McNutt T . Dose calculations: collapsed cone convolution superposition and delta pixel beam. Philips White Paper No 4535 983 02474. Eindhoven, The Netherlands: Philips Medical Systems; 2002.

[18] Yartsev S , Muren LP , Thwaites DI . Treatment planning studies in radiotherapy. Radiother Oncol. 2013;109(3):342–43. 24315361 10.1016/j.radonc.2013.11.008

[19] Trotti A and Gillison M . RTOG 1016: Phase III trial of radiotherapy plus cetuximab versus chemoradiotherapy in HPV‐associated oropharynx cancer. 2014 Accessed 2015 10/02. Available from: https://www.rtog.org/ClinicalTrials/ProtocolTable/StudyDetails.aspx?study=1016

[20] Deasy JO , Moiseenko V , Marks L , Chao KS , Nam J , Eisbruch A . Radiotherapy dose‐volume effects on salivary gland function. Int J Radiat Oncol Biol Phys. 2010;76(3 Suppl):S58–S63. 20171519 10.1016/j.ijrobp.2009.06.090 PMC4041494

[21] Rancati T , Schwarz M , Allen AM , et al. Radiation dose‐volume effects in the larynx and pharynx. Int J Radiat Oncol Biol Phys. 2010;76(3 Suppl):S64–S69. 20171520 10.1016/j.ijrobp.2009.03.079 PMC2833104

[22] Blanco AI , Chao KS , El Naqa I , et al. Dose‐volume modeling of salivary function in patients with head‐and‐neck cancer receiving radiotherapy. Int J Radiat Oncol Biol Phys. 2005;62(4):1055–69. 15990009 10.1016/j.ijrobp.2004.12.076

[23] Dijkema T , Raaijmakers CP , Ten Haken RK , et al. Parotid gland function after radiotherapy: the combined Michigan and Utrecht experience. Int J Radiat Oncol Biol Phys. 2010;78(2):449–53. 20056347 10.1016/j.ijrobp.2009.07.1708 PMC2889151

[24] Van't Riet A , Mak AC , Moerland MA , Elders LH , van der Zee W . A conformation number to quantify the degree of conformality in brachytherapy and external beam radiation: application to the prostate. Int J Radiat Oncol Biol Phys. 1997;37(3):731–36. 9112473 10.1016/s0360-3016(96)00601-3

[25] Feuvret L , Noël G , Mazeron JJ , Bey P . Conformity index: a review. Int J Radiat Oncol Biol Phys. 2006;64(2):333–42. 16414369 10.1016/j.ijrobp.2005.09.028

[26] Bezjak A . RTOG 0813: Seamless Phase I/II study of streotactic lung radiotherapy (SBRT) for early stage, centrally located, non‐small cell lung cancer (NSCLC) in medically inoperable patients. 2013 Accessed 2013 10/07. Available from: http://www.rtog.org/ClinicalTrials/ProtocolTable/StudyDetails.aspx?study=0813

[27] Craft D , Halabi T , Bortfeld T . Exploration of tradeoffs in intensity‐modulated radiotherapy. Phys Med Biol. 2005;50(24):5857–68. 16333160 10.1088/0031-9155/50/24/007

[28] Janssen T , van Kesteren Z , Franssen G , Damen E , van Vliet C . Pareto fronts in clinical practice for Pinnacle. Int J Radiat Oncol Biol Phys. 2013;85(3):873–80. 22901383 10.1016/j.ijrobp.2012.05.045

[29] Craft D and Bortfeld T . How many plans are needed in an IMRT multi‐objective plan database? Phys Med Biol. 2008;53(11):2785–96. 18451463 10.1088/0031-9155/53/11/002

[30] Ottosson RO , Engstrom PE , Sjöström D , et al. The feasibility of using Pareto fronts for comparison of treatment planning systems and delivery techniques. Acta Oncol. 2009;48(2):233–37. 18752085 10.1080/02841860802251559

