# Supplementary figures and images for: Initial evaluation of automated treatment planning software
Source: J Appl Clin Med Phys. 2016 May 8;17(3):331–46. doi: 10.1120/jacmp.v17i3.6167 (PMC5690942; doi:10.1120/jacmp.v17i3.6167)

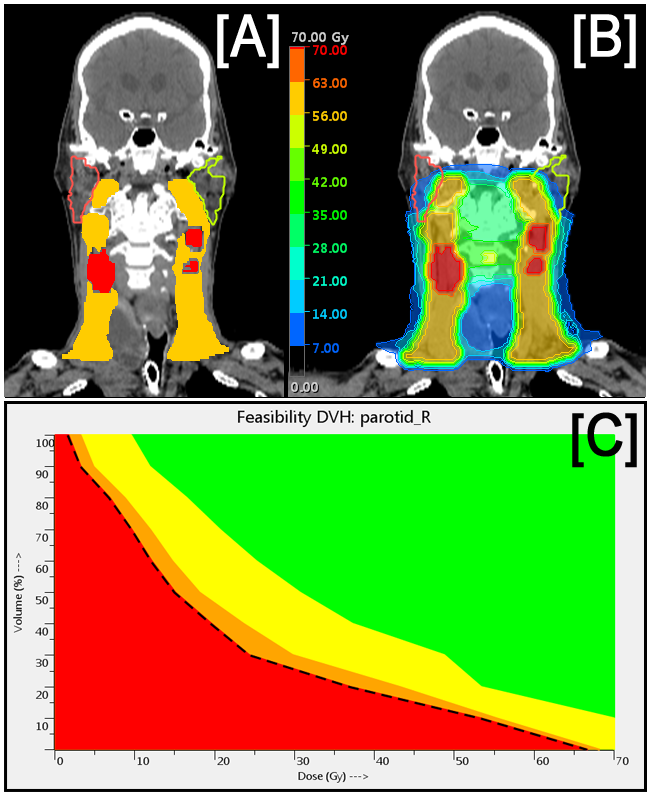

Supplement: Supplementary file 1 — Supplementary Material [file ACM2-17-331-s001.png]

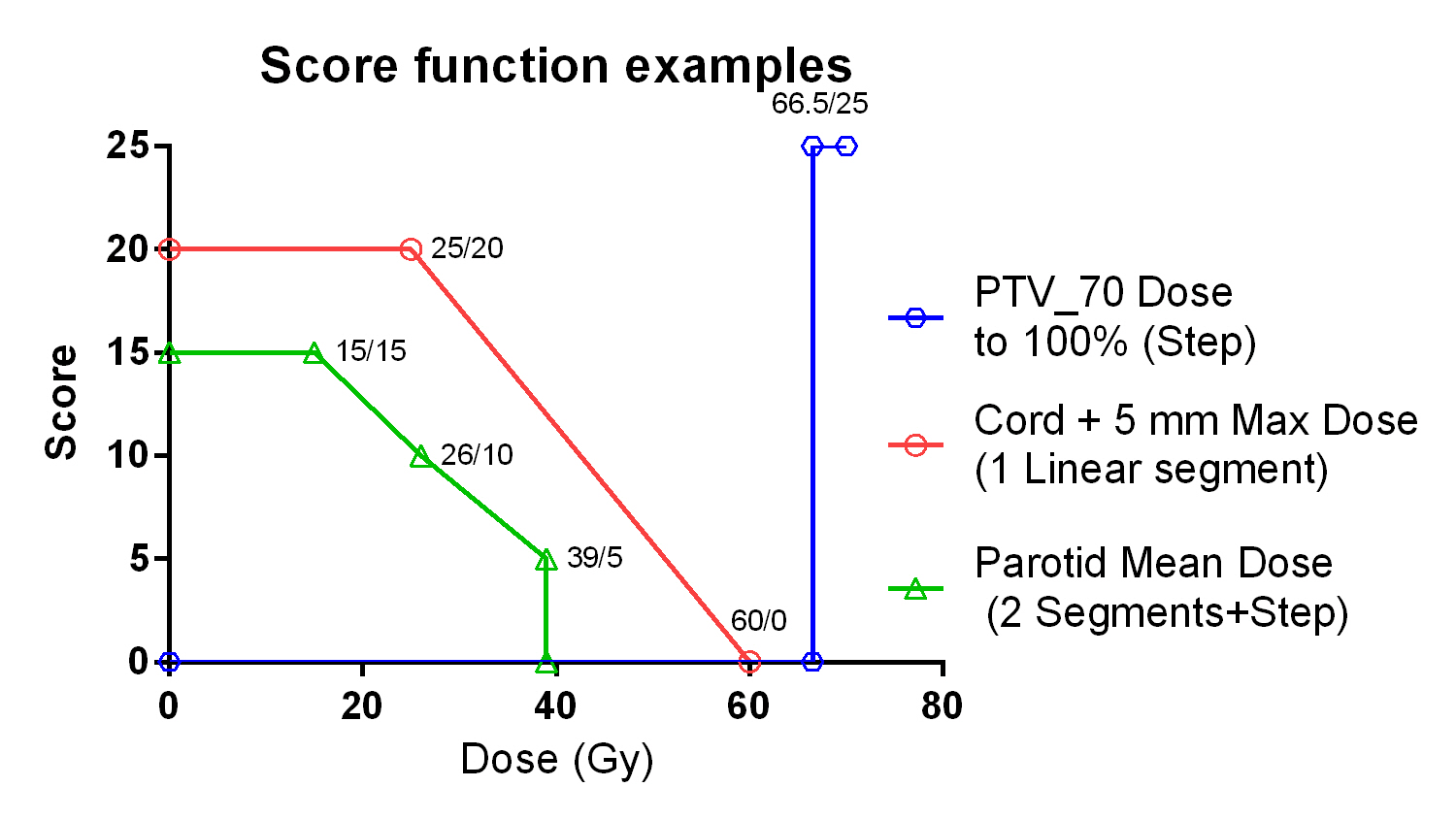

Supplement: Supplementary file 2 — Supplementary Material [file ACM2-17-331-s002.jpg]
